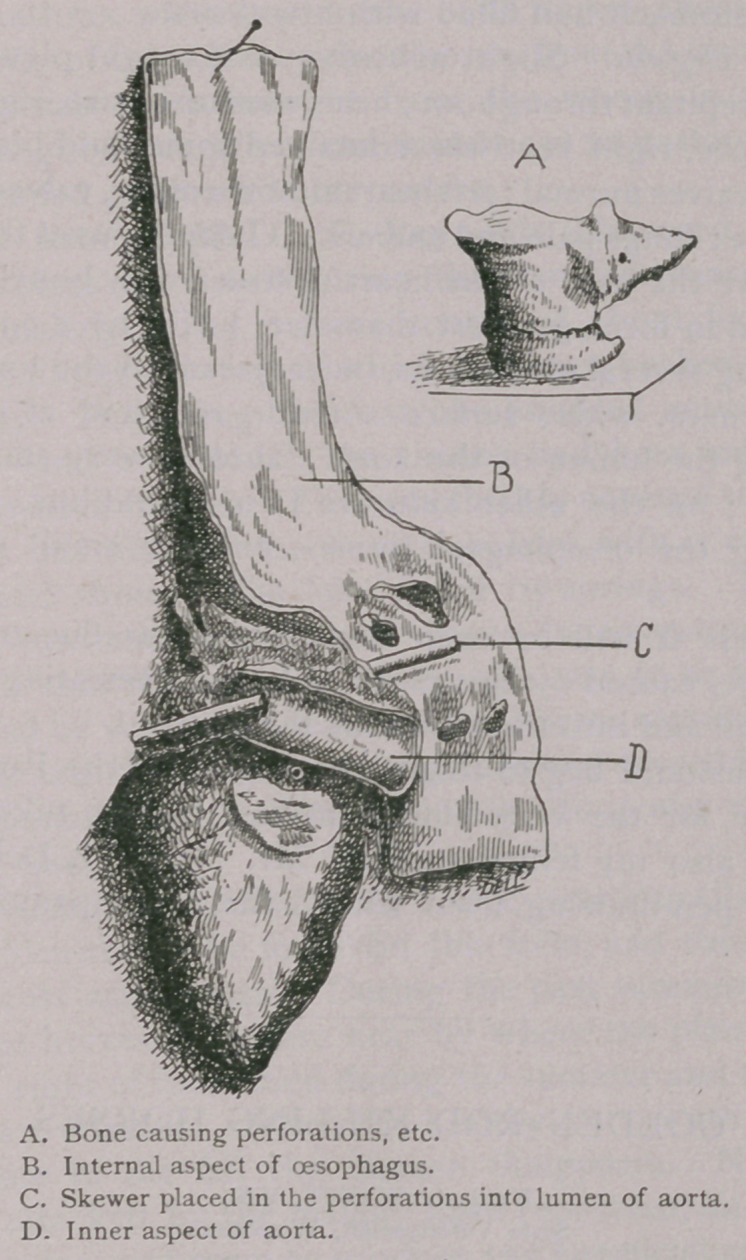# Peculiar Fatal Result of an Œsophageal Obstruction1Read before the Montreal Veterinary Medical Association.

**Published:** 1896-02

**Authors:** E. B. Thurston


### REPORTS OF CASES.

### PECULIAR FATAL RESULT OF AN ŒSOPHAGEAL OBSTRUCTION.[1]

BY E. B. THURSTON.

THE subject was an Irish terrier pup, about eight months old, brought to the hospital at 9 P.M., October 31, 1895, suffering intense pain. The history of the case was very brief, the animal being first noticed to be ill shortly after dinner the day previous.

The symptoms pointed to some gastric or intestinal trouble, the patient looking repeatedly around to one side, and tucking his nose close up against the abdomen; this would be followed by occasional efforts to vomit, a complete stretching out of the body, all four limbs being extended, and the animal lying with the abdomen to the floor; pulse very rapid; temperature 104° F.

After a hot bath, a dose of castor oil was administered and an enema of hot water and soap given. A large linseed poultice was then applied to the abdomen; this being removed every hour. Shortly after the first poultice was applied, there being no abatement in the suffering, an anodyne was administered, being repeated at intervals.

At 3 P.M. he passed both urine and feces, the latter thin, of a yellowish color, containing traces of mucus; the last opiate was given at 8 P.M.; the poultice was changed several times up to midnight, when the patient was given three ounces of beef tea and left for the night, being perfectly conscious and apparently free from pain. Both food and water were offered him, but he showed no inclination to partake of either. When seen at 1 A.M. the following morning he was sleeping quietly, but about two hours later he commenced to moan and show further signs of pain, though to all appearance it was not so severe as that of the day previous. The same treatment was continued throughout the day, namely poultices and anodynes, with an occasional enema of warm water. At 6 P.M. he was seized with a shivering fit, as if suffering from cold; although the atmospheric temperature of the quarters in which he was confined was fairly

[1] Read before the Montreal Veterinary Medical Association.

high, the patient's temperature had fallen to 98° F. However, he was wrapped in warm woollen cloths and placed in a basket near the steam radiator in the pharmacy, and an alcoholic stimulant given. An effort was made to repeat this in an hour, but power of deglutition seemed to be lost.

A. Bone causing perforations, etc.
B. Internal aspect of œsophagus.
C. Skewer placed in the perforations into lumen of aorta.
D. Inner aspect of aorta.

At 8 P.M. he was seen to be rapidly sinking, mucous membrane of the mouth was extremely pale, and the power of locomotion almost entirely gone; at 9.15 P.M. there was a profuse rectal hemorrhage, and death followed within an hour.

*Post-mortem.* Being unable to communicate with the owner, the autopsy was not held until sixty hours after death. An opening was made along the median line and abdominal organs exposed. The spleen was in its normal situation, somewhat elongated, thin and soft, of a pale-red color; on section soft and pale; pulp normal in amount, fibrous tissue normal in quantity, Malpighian bodies distinctly seen, large vessels containing well-formed clots, non-adherent; liver pale and friable. The intestines contained a blackish-green semifluid mass, intestinal mucosa pale, stomach half filled with blood-cast.

*Thoracic Organs.* Slight adhesion of the right pleura; lungs pale and crepitant throughout; heart normal in size, right auricle almost empty, right ventricle contained some fluid blood and a dark clot; valves normal; left heart almost empty, valves normal; muscle pale; lungs pale and anæmic. Œsophagus at the level of 1 c.m. above the base of the heart obstructed by bone, irregular and jagged in form, greatest diameter 3 c.m. by 2 c.m. by 1½ c.m., causing several ulcerations, one adjacent to the aorta, 2 c.m. long by 7 m.m. wide; surface smooth, red, and at one point penetrating the lumen of the aorta; another very small one is beneath it; on the other side are two ulcerations, one quite penetrating the œsophageal tissues, another small one more superficial.

From this it will be seen that death was due to internal hemorrhage, caused by this piece of bone penetrating the œsophageal wall and into the lumen of the aorta.

In conclusion, I beg to thank Dr. Martin, of the Pathological Laboratory, for the very kindly interest he has taken in this case; and also my fellow-student, Mr. Harri H. Dell, for the admirable pen-drawing from which the accompanying cut is taken.

## Figures and Tables

**Figure f1:**